# Editorial Seminars in MSK Radiology

**DOI:** 10.5334/jbr-btr.1468

**Published:** 2017-12-16

**Authors:** Paolo Simoni, Filip Vanhoenacker

**Affiliations:** 1 HUDERF, BE; 2 AZ Sint-Maarten Duffel-Mechelen and University (Hospital) Antwerp/Ghent, BE

**Keywords:** Education, Diagnotic Imaging, Musculoskeletal bone, Musculoskeletal joint

Dear participants and readers of the JBSR,

This special volume of the Journal of Belgian Society of Radiology is dedicated to the scientific program of the fourth edition of the “Seminar in Musculoskeletal Radiology” to be held in Brussels on December 15–16, 2017 (www.mskradiology.be).

We wish to express on behalf of the whole faculty a warm welcome to all delegates of this annual meeting.

The congress is held under the auspices of the Belgian Society of Radiology (BSR) and the European Society of MSK Radiology (ESSR) and has been granted with 0.9 points (CAT 3) for the E.S.S.R. Diploma.

Compared to the three previous editions, this year’s symposium will take place in a new location, the “Bowling Stone”, in Wemmel, Brussels, a modern meeting center equipped with cutting-edge audio and video facilities. The venue is easily accessible by car and from Brussels National Airport. The meeting center is very close to the Expo area of Brussels and the Atomium, a seminal totem in the skyline of Brussels and one of the prominent symbols of the city.

In keeping with the tradition of “Seminars in MSK radiology”, an outstanding national and international faculty will deliver a variety of state-of-the-art and clinically oriented lectures for radiologists and other specialists with special interest in MSK imaging. We are particularly happy to welcome leading musculoskletal radiologists from different European countries, emphasizing the European dimension of this annual meeting.

The fourth edition of this symposium will focus on imaging of the upper and lower limbs respectively, with special attention to ultrasound and interventional procedures. Other sessions will be dedicated to arthritis and trauma.

The category “Ethics and economy” comprises an important part of this meeting and covers subjects which are relevant for MSK radiologists.

Probably, the most exciting novelty of this year’s edition is the introduction of a separate “Young Stars Session” devoted to young radiologists. During this session, promising talents in MSK radiology have the opportunity to share their work, which were carefully selected by the scientific board after submission of an abstract for an original work or a pictorial review. This initiative will certainly boost their enthusiasm and interest in MSK radiology. Indeed, young talents form the backbone of the future of our subspecialty discipline.

We would like to express our deepest gratitude to all speakers who provided a well-written manuscript on the contents of their lectures, resulting in an issue of JBSR that may serve as very useful reference text in our daily clinical practice. A special word of thanks goes to the editor-in-chief of the JBSR, Dr. A. Nchimi, for giving us the opportunity to distribute this special printed edition of the journal for the first time since the start of this annual symposium.

Apart from the scientific program, the city of Brussels offers so many attractions, including the marvelous historical Grand Place, the Peeing Boy (Manneken Pis), the Atomium, its many museums such as Magritte Museum etc., and promenades around the “Art Nouveau” quarters. Moreover, Brussels during the wintertime has a very special Christmas atmosphere, which make strolling through the city and shopping an unforgettable experience.

We wish you a very pleasant stay in Brussels, a fruitful educational experience, and a nice reading of this JBSR issue.

Prof. Paolo Simoni,

President of the Congress

Prof. Filip Vanhoenacker,

President of European Society of Skeletal Radiology (ESSR)(2018)

